# Estimation of Pedestrian Pose Orientation Using Soft Target Training Based on Teacher–Student Framework

**DOI:** 10.3390/s19051147

**Published:** 2019-03-06

**Authors:** DuYeong Heo, Jae Yeal Nam, Byoung Chul Ko

**Affiliations:** Department of Computer Engineering, Keimyung University, Daegu 42601, Korea; cloudnine148@gmail.com (D.H.); jynam@kmu.ac.kr (J.Y.N.)

**Keywords:** soft-target training, teacher–student algorithm, model compression, pedestrian orientation, wavelet transform

## Abstract

Semi-supervised learning is known to achieve better generalisation than a model learned solely from labelled data. Therefore, we propose a new method for estimating a pedestrian pose orientation using a soft-target method, which is a type of semi-supervised learning method. Because a convolutional neural network (CNN) based pose orientation estimation requires large numbers of parameters and operations, we apply the teacher–student algorithm to generate a compressed student model with high accuracy and compactness resembling that of the teacher model by combining a deep network with a random forest. After the teacher model is generated using hard target data, the softened outputs (soft-target data) of the teacher model are used for training the student model. Moreover, the orientation of the pedestrian has specific shape patterns, and a wavelet transform is applied to the input image as a pre-processing step owing to its good spatial frequency localisation property and the ability to preserve both the spatial information and gradient information of an image. For a benchmark dataset considering real driving situations based on a single camera, we used the TUD and KITTI datasets. We applied the proposed algorithm to various driving images in the datasets, and the results indicate that its classification performance with regard to the pose orientation is better than that of other state-of-the-art methods based on a CNN. In addition, the computational speed of the proposed student model is faster than that of other deep CNNs owing to the shorter model structure with a smaller number of parameters.

## 1. Introduction

The pose orientation estimation (POE) of humans based on computer vision has many potential applications, including human–robot interaction [1,2], video surveillance [3], and autonomous driving [4], because it supports important visual cues of human intention and behaviour. For example, robots can move and orientate themselves to observe the user and achieve a more natural interaction [2], and surveillance cameras can characterise the activity and interactions of people in a more precise manner. In terms of autonomous driving, the pedestrian body orientation is a good indicator as to what the pedestrian will do next [4] by inferring the pedestrian’s walking direction.

There are three types of approaches to POE. Sensor-based approaches use the gyroscope sensor of a smartphone [5] or a Kinect sensor [6] because the geometry information brought about by the depth helps overcome the fundamental problems in computer vision, such as a cluttered environment, changes in illumination, and partial occlusions. However, these approaches can only be performed using a smartphone, and if the distance between the pedestrian and the sensor is too great, the recognition may be impaired.

Optical marker-based motion capture approaches [7,8,9] use marker and infra-red (IR) cameras. Optical systems offer very accurate POE because these approaches use many retro-reflective markers on the human subject at several locations on their body. Optical marker-based approaches are mainly used for movies, virtual reality, games, etc., which require precise 3D model generation, and are partially used in POE. The optical system does not work well outdoors due to the limited capture volume that is constrained by the size of the room. In addition, the user must wear the markers at a designated position on the body and installation cost of multiple IR cameras is relatively expensive than that of other sensors. Recently, several studies [10,11,12] have been conducted to capture 3D motion using only several color cameras without optical markers.

Camera-based POE approaches can be used to recognise the orientation of a pedestrian at a longer distance and estimate additional behavioural information (face, facial expressions, posture, etc.). In certain approaches, the pose orientation can be predicted using the trajectory information based on tracking. These approaches are effective in inferring a pedestrian’s intended movement when the camera is static, such as with a surveillance camera. In contrast, they are inappropriate for use in a moving camera environment, such as on a robot or in an intelligent vehicle, because they have difficulty tracking the pedestrian correctly for a certain amount of time and analysing the pedestrian’s direction. Therefore, alternative POE methods have proposed single-frame approaches in a moving camera environment [2,3,11,12]. Single-frame-based POE methods allow the heading of the pedestrian to be recovered without having to observe several frames regardless of whether the camera is moving.

Among a few applications related to POE, this study focuses on an estimation of the pedestrian’s pose orientation for collision avoidance in an advanced driver assistant system (ADAS). In an ADAS, a vehicle can detect pedestrians and predict their intentions in advance based on their POE. Therefore, the possibility of a collision is significantly reduced because the driver can be alerted if a pedestrian is stepping onto the road without noticing the vehicle. To apply POE to the realizable ADAS, various environments, such as day and night and weather, should be considered. As one solution, an RGB camera and IR camera can be used at the same time. However, it is necessary to develop different POE algorithms separately for each camera sensor or to develop an image-to-image translation algorithm for converting night-time image to day-time image as the pre-processing. However, because we aim to recognize the POE of the pedestrian in the daytime image, the experiment is carried out with the images taken from the moving vehicle during the normal daytime. Instead, we design the algorithm to be less susceptible to various illumination changes by using the handcraft wavelet transform as pre-processing.

In this paper, we present a new POE method for pedestrian tracking using a single frame captured from a vehicle-mounted moving camera and a POE classifier based on an ensemble of soft-target trained shallow neural networks and a random forest (RF). The convolutional neural network (CNN) based orientation estimation requires a large number of datasets for training and testing. Moreover, it requires a massive high-level computing device as compared to a conventional classifier. Therefore, in this study, a teacher–student framework inspired by [13] and [14] is adopted.

The original purpose of a teacher–student framework is to build a deep and wide teacher neural network with high performance based on large amounts of training data and to construct a shallower student network with an equal level of performance based on the teacher network. This idea allows the student network to capture not only the information provided by the true labels but also the finer structure learned by the teacher network [14]. The core differences between present teacher–student framework and the ones presented in [13,14] as follows.

- We propose a new soft output by combining the softened output of the teacher network and the teacher RF to obtain a softer output of the teacher model.
- The proposed approach uses an additional training dataset B to prevent the overfitting of the teacher model and a new soft target dataset $B^{*}$ is able to capture more information than the original hard target data.
- The constructed shallower student model can reduce the model size and be capable of mimicking the POE capability of the teacher model without sacrificing accuracy by combining two different classification models.

In a previous study [15], we proposed a simple teacher–student model, although the student model used only a short CNN without combining an RF. However, in this study, we first redesign the teacher–student framework to avoid reducing the network size and to allow a student network capable of outputting flexible pedestrian orientation information to be constructed by combining soft-target trained shallow networks and an RF. Second, soft weighting scheme that predicts the final pedestrian angle according to the probability of the neighbouring two classes, is proposed instead of searching for only the highest probability of each class. Moreover, we describe the successful application of the proposed method to two benchmark datasets, and we confirm that its POE accuracy is similar to or higher than that of other CNN-based related methods with shorter processing time.

**Figure 1:**
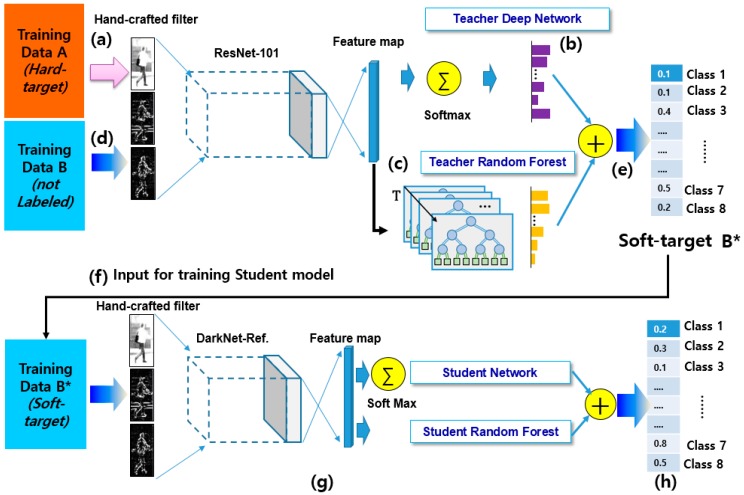
Teacher–student learning framework using hard- and soft-target data (in order of learning process): (**a**) dataset A labelled with a hard target input into the (**b**) teacher deep network and used for training teacher deep network, (**c**) teacher random forest (RF) is trained using feature maps of teacher deep network, (**d**) after finishing training of two-teacher model, unlabelled training dataset B input into the trained two-teacher model, (**e**) the soft output of the two teachers combined into one soft target vector and (**f**) a soft target dataset $B^{*}$ of the teacher network are used for training the target student network, (**g**) training of the student model composed of student network and student RF, and (**h**) the final class probability is generated by combining output probability of two-student model.

Figure 1 shows the overall structure of the proposed framework. As Figure 1 indicates, the proposed framework consists of a teacher model and a student model. A dataset A labelled with a hard target (1/0) input into the teacher deep network (Figure 1a) and used for training teacher deep network (Figure 1b). Another teacher RF is trained using feature maps of teacher deep network (Figure 1c). After finishing training of two-teacher model, unlabelled training dataset B input into the trained two-teacher model (Figure 1d) and the teacher model generates a soft-target (probability values) for each class by combining the output of the teacher network and teacher RF (Figure 1e). Then, a soft target dataset $B^{*}$ of the teacher network are used for training the target student shallow network and student RF (Figure 1f,g) and the final class probability is generated by combining output probability of two-student model (Figure 1h). Teacher and student networks use different structures. A student network that is shallower than a teacher network is only newly learned using soft-target data learned by a teacher. We use a shallow deep network as the student network instead of reducing teacher network because of its fast computational speed and learn the student network again with the soft-target dataset learned from the teacher model.

The remainder of this paper is organised as follows. In Section 2, we introduce some previous POE methods focusing on intelligent vehicles. Section 3 first describes how to construct a teacher model and a soft-target for the training data. Then, the training of a student model using a soft-target generated from a teacher model is introduced. In Section 4, we present experiments demonstrating the accuracy and applicability of our proposed POE method. Finally, some concluding remarks and areas of future research are presented in Section 5.

## 2. Related Studies

Because this paper focuses on the POE of a pedestrian in a single image captured by a moving vehicle, we do not consider 3D POE techniques using stereo cameras or RGB+Depth sensors.

Studies on automatic POE can be divided into two groups according to whether the features are handcrafted or generated through the output of a deep neural network. In conventional POE studies, the first strategy is a detector-driven approach. With this approach, a pedestrian region is detected from an input image, and various robust lower-level image features, such as a histogram of gradient or local binary pattern features, are extracted from the pedestrian region. Then, a single pre-trained POE classifier or an ensemble of POE classifiers, such as a support vector machine (SVM), AdaBoost, and RF, conduct the orientation estimation using the extracted features. Shimizu and Poggio [16] estimated the walking direction of pedestrians from multiple still images by classifying each image of a walking sequence using an SVM and combining the outputs of the classifier. The final direction is then inferred through a majority vote among the classifications of each image in the sequence. Enzweiler and Gavrilar [11] presented an integrated method of pedestrian classification and orientation estimation using a single frame. Pedestrian classification is conducted based on a maximum-a-posteriori decision between the pedestrian and non-pedestrian class, and the POE is inferred by approximating the probability density in terms of a set of view-related models of the pedestrian’s body orientation. Flohr et al. [4] proposed a head and body orientation detection method in which anatomical constraints are estimated and integrated over time through particle filtering. In their study, they used data from a vehicle-mounted stereo-vision camera in a realistic traffic setting and proved that a joint probabilistic orientation estimation framework reduces the error in the mean absolute head and body orientation.

Another POE strategy is a template (model) driven approach using an appearance template, active shape model, or active appearance model applying a human shape model as an alternative to a data-driven approach [4]. A statistical shape model [17] uses a multimodal shape representation involving a set of distinct linear space models that correspond to clusters of similar body shapes. This shape model is used to detect pedestrians with various poses based on a well-behaved matching criterion involving a multi-feature distance transform.

Orozco et al. [12] proposed a head pose descriptor using similarity distance maps by indexing each pixel of a head image to the mean appearance templates of head images of different poses. These distance feature maps are then used to train a multi-class SVM for pose classification. In addition, regression- and manifold-based methods have also been proposed. Refer to [18] for a detailed description of such approaches.

The main advantage of conventional POE approaches is that they require relatively less computing power and memory than deep-learning-based approaches. However, their feature extraction and classifiers should be designed by programmers, and they cannot be jointly optimised to improve the performance [19].

Many deep-learning-based POE algorithms have also been recently studied under human-activity recognition scenarios. Choi et al. [2] proposed the use of a lightweight CNN for estimating the human body orientation to allow a robot to move towards a user’s frontal plane. This method does not apply a pooling layer because the POE requires more spatial information than in object recognition. Hara [20] compared three continuous orientation prediction approaches designed for a deep CNN. According to the experiment results, a discretisation-based approach that converts the continuous orientation into a set of discrete orientations, and then converts the discrete orientation outputs back to a continuous orientation using a mean-shift algorithm, achieves state-of-the-art performance. Lee et al. [18] proposed the use of a convolutional random projection network by combining a CNN with an RF. This method uses rich filter banks that capture the filter responses. These responses are used to generate compressive sensing and are applied randomly to each node of a tree as a split function to learn the discriminative filters in each rectangular region. Raza et al. [21] used appearance-based POE prediction by employing a deep-learning mechanism consisting of a hierarchical structure containing several types of layers attached one after the other. This method trains the networks separately on two prepared datasets with eight orientation bins.

Unlike conventional POE approaches, deep-learning-based POE approaches tend to completely remove or highly reduce the dependence on physics-based models and/or other pre-processing techniques by enabling ‘end-to-end’ learning directly from the input image [19]. However, these approaches still require the collection of a large amount of training data as well as a more massive higher-level computing device than convention approaches for applying the training and inference processes.

## 3. Teacher–Student Framework

As mentioned in Section 2, deep-learning networks require large numbers of parameters to generate a deep model. Therefore, they need large amounts of memory and time to conduct a huge number of multiplications at the inference time. In order to solve this drawback of such wide and deep network models, this study adopts a teacher–student framework to construct a shallower student model with an equal level of performance based on the teacher network as shown in Figure 1.

The proposed teacher–student framework is used not only to reduce the network size but also to construct a student network capable of mimicking the POE capability of the teacher model.

### 3.1. Training of Teacher Model

The original teacher model is constructed using a deep and wide teacher network as well as a wide teacher RF with a high level of performance based on a large amount of training data. The teacher model is first trained using a labelled dataset A consisting of 0 or 1 (hard target).

In [14], the teacher network T applies a softened softmax function (1) to the vector of the teacher pre-softmax activations, aT, to obtain a soft-target (output probability) differently from the softmax of the general CNN model. The basic idea of the teacher is to allow the student network to capture not only the information provided by the true labels but also the finer structure learned by the teacher network:

(1) $$P_{T}^{\tau }=\frac{\exp\left(\frac{{\mathrm{aT}}_{i}}{\tau }\right)}{\sum_{j}\exp\left(\frac{{\mathrm{aT}}_{j}}{\tau }\right)}$$

where aT might be extremely close to the one hard target representation of the sample’s true label, whereas the teacher’s softened output ($P_{T}^{\tau }$) has a softer distribution as the temperature (τ > 1) increases.

This method can soften the signal arising from the output of the teacher network and provide more information to the student network during the training of the student model. However, because the performance of the student network is sensitive to the temperature (τ), and this value has to be determined empirically for all training data, estimating the optimal τ requires significant effort.

To reduce the effort required in determining τ and obtain a soft output of the teacher model, we chose a new soft output by combining the softened output of the teacher network and the teacher RF, as shown in Figure 1. An RF is a decision tree ensemble classifier that is known to process very large amounts of data with high training speeds compared to conventional classifiers [22]. Moreover, an RF essentially presents a softer distribution of the classification results for the particular class.

In detail, we first train the teacher network using a training dataset $A=\left\{\left(x_{i},y_{i}\right)|i=1,2,\ldots N\right\}$ consisting of N hard targets with M dimensional input vector $x_{i}=\left(x_{i1},x_{i2},\ldots ,x_{\mathrm{iM}}\right)$ and a scalar class label $y_{i}=\left\{g_{1},g_{2},\ldots ,g_{C}\right\}$ marked by an expert for $x_{i}$.

The teacher network is generated based on the residual network (ResNet)-101 model [23] with class-labelled (hard targets) training data. The structure of the teacher network has 101 parameter layers, one average pooling, followed by one fully connected layer. The baseline architectures are the same as in a normal CNN, although ResNet adds a shortcut connection (skipping one or more layers) to each pair of 3 × 3 filters. Moreover, ResNet uses identity mapping for all shortcuts and zero-padding to increase the number of dimensions. The outputs of the short connections are added to the outputs of the stacked layers.

Given training dataset A, we fine-tune a ResNet-101 model pre-trained on ImageNet to the smaller dataset A to update all of the network weights for the new task. After training the teacher network, the softened softmax function (1) provides a soft output on eight output units (classes).

As the second classifier, an individual decision tree of the teacher RF is trained using the output vector of the last feature maps for input vector $x_{i}$ with its hard class label $y_{i}$. The training of the decision tree is based on a random sampling of the subset and split function selection using information gain [23]. The final class distribution of a sample **x** is generated using the ensemble (arithmetic averaging) of each distribution $p_{t}\left(c_{i}|x\right)$ of all trees T:

(2) $$p\left(c_{i}|x\right)=\frac{1}{T}\sum_{t=1}^{T}p_{t}(c_{i}|x)$$

The final procedure of the training teacher model is to combine the softened softmax and RF output to produce a soft target, as shown in Figure 1e.

After completing the training of the teacher model, another much larger and non-labelled training dataset B is applied to the teacher model, and a new dataset $B^{*}$ consisting of soft targets (class probability) as opposed to a hard target is constructed.

Unlike the algorithm in [14], which creates a soft target set by applying only one dataset A to the teacher model, the proposed approach uses an additional training dataset B to prevent the overfitting of the teacher model. A new soft target dataset $B^{*}$ is able to capture more information than the original hard target data by retaining the class relationship between the different classes [24,25]. Moreover, we can obtain more flexible classification results than using a hard target dataset.

After all *M* samples x included in dataset B have been trained, a new dataset $B^{*}$ transcribed with a class probability $p_{i}^{*}$ (soft target) is constructed:

$$B^{*}=\left\{\left(x_{i},\;p_{i}^{*}\right)|i=1,2,\ldots M\right\}$$

A difference in the recognition performance can also occur depending on the number of decision trees used in the RF, and we set the number of trees to 300, as described in [26].

### 3.2. Handcrafted Filters

In addition to the softened softmax function, we feed the model with three handcrafted filter responses of a wavelet transform, namely, the coefficients of two high-pass filtered sub-images (LH and HL) and one low-pass filtered sub-image (LL), using the Daubechies D4 wavelet [27] as the input along with a grey image (Figure 1a), because feeding the appropriate handcrafted features can lead to improved results for certain classification problems [28]. Moreover, because a wavelet transform has a good spatial frequency localisation property and can preserve both the spatial information and gradient information of an image, it is helpful in improving the POE performance in various lighting conditions. The improvement in the recognition performance by a wavelet transform is described in detail in Section 4.

### 3.3. Training of Student RF

After training the teacher model, we construct a student model to derive a flexible orientation of the pedestrian using the soft target dataset $B^{*}$ generated from the teacher model. The student model is composed of one student network and one student RF, as in the teacher mode.

The student network is generated by modifying the DarkNet reference model [29] instead of reducing ResNet-101 model [23] because the computational speed of DarkNet reference is 16 times as fast as ResNet-101 and twice as fast as AlexNet [30] and on a CPU with 1/5th and 1/10th the number of parameters. Therefore, instead of compressing the teacher network, we use a shallow DarkNet reference model as the student network and learn the student network again with the soft-target dataset learned from the teacher model.

The structure of the student network consists of eight convolution layers with seven max-pooling layers and one average pooling layer following each convolution layer. The front seven convolutional layers have 3 × 3 sized convolution filters with a max pooling layer of 2 × 2 sized filters, and the last convolutional layer has 1 × 1 sized convolution filters with an average pooling layer instead of a fully connected layer to avoid an overfitting problem and to reduce the number of trainable parameters of the fully connected layer. In addition, batch normalisation is applied to each convolutional layer and leaky ReLU [31] (LReLU) is used as an activation function to fix the dying ReLU problem. The LReLU function f(x) has a small negative value when x < 0 instead of the function being zero.

(3) f(x)=max(0.01x,x)

To train the student network, we use convolutional weights pre-trained on ImageNet [32] and conduct the fine-tuning using the soft-target dataset $B^{*}$. The cross-entropy criterion is based on frame-wise minimisation [24] by replacing the hard target vector with a soft-target vector as follows:

(4) $$ℒ\left(W_{S}\right)=-\overset{N}{\underset{i=1}{\sum }}\overset{C}{\underset{j=1}{\sum }}P_{T}(x_{i}|c_{j})\log P_{S}(x_{i}|c_{j})$$

where *N* is the number of samples in training set $B^{*}$, and *C* is number of classes. In addition, $P_{T}(x_{i}|c_{j})$ and $P_{S}\left(x_{i}|c_{j}\right)$ are the posterior class probability of the teacher and student for input vector $x_{i}$, respectively. Algorithm 1 describes the brief training procedure of a student network.
**Algorithm 1**: Student network training$B^{*}$: Soft target dataset$W_{S}$: Parameters of a student network1. Initialise $W_{S}$
2. Input $B^{*}$ to pretrained network3. Compute loss function $ℒ\left(W_{S}\right)$ using Equation (4)4. $W_{S}^{*}\leftarrow \arg\underset{W_{S}}{\min}ℒ\left(W_{S}\right)$
5. Select the best parameter $W_{S}^{*}$ for student network


Similar to a student network, the initial decision trees of the student RF use the same structure as the teacher RF, such as the number of trees, tree depth, and split function with the split threshold of each node of an individual tree.

The training of the decision tree of the student RF uses input vector v and class probability $p_{i}^{*}$. The input vector has 128 dimensions generated from the last feature maps (4 × 4 × 8), and the class probability is estimated from the output of the teacher model. From the collected training data consisting of an output vector with the class probabilities of soft target dataset $B^{*}$, a decision tree of the student RF randomly selects p variables with class probabilities from the samples. Let us allow $B_{O}^{\prime }$ to represent the samples at node *O*. The pre-trained and randomly generated split functions $f\left(v_{p}\right)$ iteratively split the random subset $B_{O}^{\prime }$ into left (${B^{\prime }}_{l}$) and right (${B^{\prime }}_{r}$) subsets at node *O*. To select the best split function, the entropy E(O) of node O is estimated using only p variables with probability distribution $P_{j}^{*}$. In this paper, we define the entropy $E\left(B_{O}^{\prime }\right)$ of node O, as inspired by [26], as follows:

(5) $$E\left(B_{O}^{\prime }\right)=-\overset{N}{\underset{i=1}{\sum }}\overset{C}{\underset{j=1}{\sum }}P_{j}^{*}\left(x_{i}\right)\log P_{j}^{*}\left(x_{i}\right)$$

Using the same method, the left and right subsets at node *O* are divided into $B_{l}^{\prime }$ and $B_{r}^{\prime }$, and entropy $E\left(B_{l}^{\prime }\right)$ and $E\left(B_{r}^{\prime }\right)$ are computed. From three entropies, the information gain ΔE of node *O* is calculated using Equation (6).

(6) $$\Delta E=E\left(B_{O}^{\prime }\right)-\frac{\left|B_{l}^{\prime }\right|}{\left|B_{O}^{\prime }\right|}E\left(B_{l}^{\prime }\right)-\frac{\left|B_{r}^{\prime }\right|}{\left|B_{O}^{\prime }\right|}E\left(B_{r}^{\prime }\right)$$

This process is repeated during the application of the number of candidate split functions, and we determine the best split function $f\left(v_{p}\right)$ for node O as the function having the maximum ΔE.

After the initial decision tree ${\mathrm{Tr}}_{t}$ has been expanded, the probability distributions on C classes are stored in the leaf nodes. We then estimate the cross-entropy in Equation (6), with $P_{ij}^{*}\left(Te\right)$ representing the *j*-th class distribution of sample *i* of the $B^{*}$ dataset transcribed by the teacher RF, and $P_{ij}^{*}\left(S_{t}\right)$ representing the *j*-th class distribution of sample *i* based on the constructed decision tree *t*. The general form of the final cross-entropy is

(7) $$\mathrm{Tr}{\left(\mathrm{Te},S\right)}_{t}=-\overset{N}{\underset{i=1}{\sum }}\overset{C}{\underset{j=1}{\sum }}P_{ij}^{*}\left(Te\right)\log\left(P_{ij}^{*}\left(S_{t}\right)\right)$$

Motivated by the impressive performance of a boosted RF [33,34], we repeat the boosting to update the t-th weak decision tree until the $\mathrm{Tr}{\left(\mathrm{Te},S\right)}_{t}$ is below the minimum criteria θ. When T random decision trees are completed, the student RF finally becomes T trees composed of the probability distributions per class. Algorithm 2 describes the procedures used in the student RF training.
**Algorithm 2**: Student RF training$B^{*}$: Soft target datasetT: The maximum number of decision trees to grow$T-RF_{t}$: t-th tree structure of teacher RF$S-RF_{t}$: t-th tree structure of student RFθ: The minimum threshold for stopping the boosting 1. Copy $T-RF_{t}$ to $S-RF_{t}$ (transfer learning)2. Input $B^{*}$ with v to one of the decision trees of $S-RF_{t}$
3. Generate split functions f(v) at node O4. Compute information gain ΔE of node O using Equation (6)5. Compute the entropy $\mathrm{Tr}{\left(\mathrm{Te},S\right)}_{t}$ using Equation (7)6. If the cross-entropy is below θ
$\mathrm{If}\;\mathrm{Tr}{\left(\mathrm{Te},S\right)}_{t}<\theta$6-1. then save current $S-RF_{t}$t=t+1, go to step 16-2. otherwise, go to step 2.7. Continue from step 1 to 5 until T trees are constructed.


### 3.4. Pedestrian Orientation Estimation

**Figure 2:**
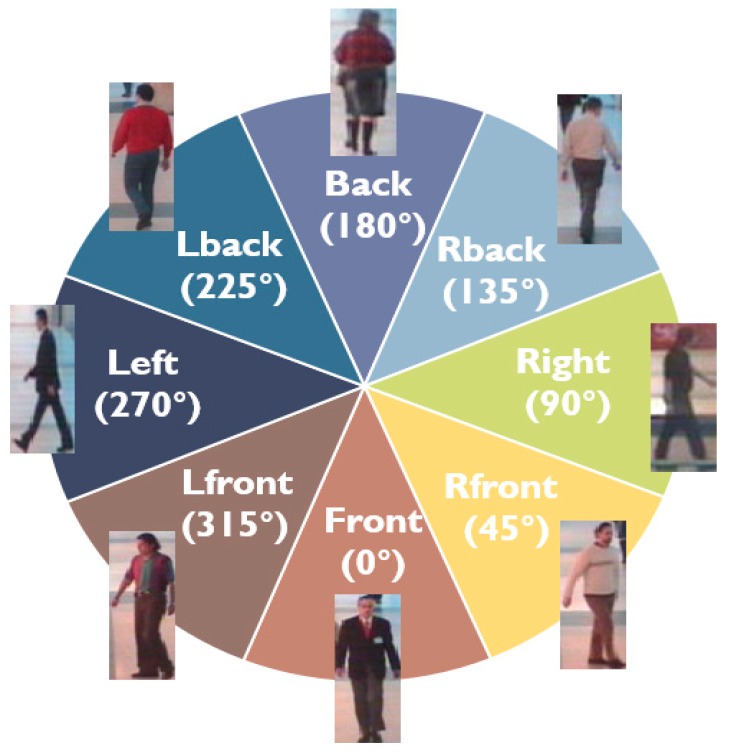
Eight orientations recognized by the proposed system and corresponding examples of pedestrian poses.

In predicting the orientation of the pedestrians, the number of classes increases when every orientation is predicted. Therefore, most existing studies [1,2,3,4,5,6,12] have used a method for recognising the orientations by dividing them into N clusters as shown in Figure 2. For example, the TUD dataset consists of 5228 images of pedestrians with a bounding box and orientation annotations, such as back, front, left, right, left back, right back, left front, and right front. For the TUD dataset, the orientation classes are divided into 45 degrees. However, ambiguity occurs because pedestrians cannot clearly judge a certain angle. Therefore, it is inappropriate to express the angle of all pedestrians as zero or 45 degrees, or to use expressions such as ‘front’ and ‘back’. Apart from previous POE approaches, we propose a soft weighting method using the final class probability (Figure 1h) and neighbouring combination. First, the final probability is generated by combining the output values of the deep network and RF to estimate any ambiguous pedestrian angles more accurately.

Instead of searching for only the highest probability of each class, soft weighting first finds the angle class ($c_{k}$) with the highest probability and predicts the final pedestrian angle according to the probability of the neighbouring (back and forth) two classes ($c_{k-1},c_{k+1})$. Then, the weight of the maximum probability class $w\left(c_{k}\right)$ is estimated to obey the following exponential weighting scheme.

(8) $$w\left(c_{k}\right)=1/\left(\exp(F\cdot P\left(c_{k}\right)\right)$$

where $P(c_{k})$ is the probability of *k*-th class $c_{k}$. Weighting factor *F* can be chosen to maximise (minimise) the influence of $P\left(c_{k}\right)$ on w(·). When *F* equals 1, a change in $P\left(c_{k}\right)$ will be exponentially reflected in w(·). The exponential weighting is more sensitive to changes in the local feature relevance, and results in a greater performance improvement [35]. In this study, we set *F* to 0.5 in accordance with the experiment results.

Equation (8) is also applied to the probability values of the forward ($c_{k+1}$) and backward ($c_{k-1}$) classes based on the class having the maximum probability. After the three classes and their respective weights are determined, the final pedestrian orientation $\tilde{O}$ is determined using

(9) $$\tilde{O}=\frac{1}{3}\overset{k=1}{\underset{k=-1}{\sum }}w\left(c_{k}\right)\cdot O_{k}$$

Based on Equation (9), the final pedestrian direction is not determined as one of the N directions but is instead adjusted to have a value close to the direction of the actual pedestrian.

## 4. Experiment Results

In this section, we evaluate the performance of the proposed method using a benchmark database and demonstrate the effectiveness of the proposed method through a comparative experiment using other approaches described in recent studies. In the experiment, we first verify the performance of the POE to prove that the proposed algorithm is effective in terms of the orientation prediction of various pedestrian poses.

The experiment was conducted using an Intel Core i7 processor with 24 GB of RAM running Microsoft Windows 10. In addition, all RF approaches, including the teacher-RF and student-RF, were executed based on the CPU, and the deep teacher network was executed using a single Titan X GPU. In addition, we used DarkNet framework [29] to train ResNet-101 model [23] and DarkNet reference model. Teacher and student-RF were implemented by C++ code without using a library. In terms of training time, ResNet101 took approximately 7 h and DarkNet reference took approximately 2.5 h on average using the same GPU, and RF took about 3~7 min using only the CPU and same database.

**Figure 3:**
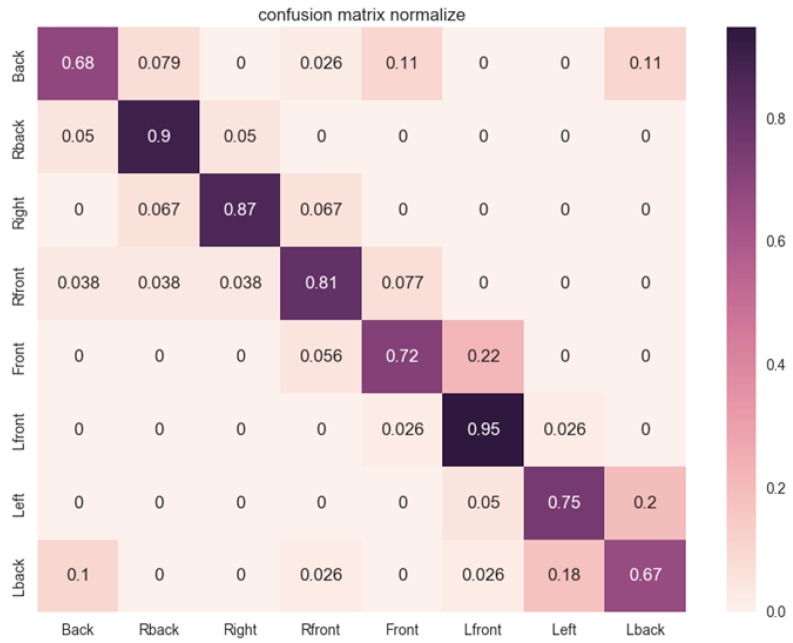
Confusion matrix based on accuracy (ACC) of proposed method (%).

For the training of the deep teacher network, the batch size, momentum, learning rate, and weight decay were set to 32, 0.9, 0.001, and 0.0005, respectively. For the RF, the important parameters in terms of the performance and memory required to store the trees are the depth of the trees and their number. In our experiments, we chose a maximum tree depth of 20 and set the number of trees of the teacher RF to 300 according to [26]. To determine the number of trees of the student RF, we sequentially decreased the desired number of trees to 250, 200, 150, 100, 70, and 50, and set the value to 70 for better accuracy and a faster computational time, as described in the results of Figure 3. The student network, described in Section 3.3, and the student RF were re-trained using a soft target training set $B^{*}$ based on Algorithm 1; Algorithm 2.

Although many datasets related to pedestrian detection are available, relatively few have evaluated the orientation of the pedestrians. Therefore, this study carried out POE experiments on the most popular TUD multi-view pedestrian dataset [36], which consists of 5228 images of pedestrians with a bounding box and discrete orientation annotations. The dataset contains 4732 full-body pedestrian images for training, and 248 for validation and 248 for testing. The images in the TUD dataset were captured under real-world street conditions, and all images contain a large variety of poses and clothing, making this dataset much more challenging [20]. It is common knowledge that models trained with small datasets do not generalise the data from the validation and test set well, resulting in an overfitting [37]. To reduce an overfitting, we increased the size of the dataset by applying data augmentation, such as image shifting, zooming in or out, rotating using a random angle between −15 and +15 degrees, left–right flipping, and cropping. All training images include original and duplicate images fed into the teacher model. According to the aforementioned data augmentation, we assigned 4,732 images to training dataset A and 4732 images to training set B.

As the second dataset, we used the KITTI dataset [38], which is a challenging real-world computer vision benchmark that includes stereo imaging, an optical flow, visual odometry, 3D object detection, and 3D tracking. Among nine different categories available, we conducted our experiments on the pedestrian category. We divided the pedestrian category of the KITTI dataset into a training set consisting of 5415 images and a validation set consisting of 2,065 images. We also increased the size of the dataset by applying data augmentation only to training set and used an overall training dataset of 4732 images. Difficulties with the dataset are divided into three levels according to their size, occlusions, and truncation levels, namely, *easy*, *moderate*, and *hard*. Detections in unimportant areas or detections smaller than the minimum size do not count as false positives. For training the student model for the KTTI dataset, the training data were applied to the teacher model, and the soft target data, which are the output of teacher model, were applied to the student network and student RF. During the model training, we normalised the orientation of the pedestrian in eight angles and estimated the continuous orientation value using Equation (8) when testing.

To validate the effectiveness of the orientation estimation of the eight classes, we measured the precision, recall, and false positive rate (FPR) for the TUD dataset, which are generally used to evaluate the performance of object recognition. In addition, the accuracy (Acc) is used for an evaluation of the pose and the confusion matrices to compare the performance between classes. The accuracy is the ratio of true outcomes (both true positive to true negative) to the total number of cases examined. For the KITTI dataset, we used the average orientation similarity (AOS) because the pedestrian data of the KTTI dataset are labelled as having a continuous orientation, which differs from the TUD.

### 4.1. Performance Evaluation on TUD Dataset

We compared the performances of five state-of-the-art methods to verify the effectiveness of the proposed POE method: (1) MoAWG [39], which classifies the POE using an array of extremely randomised trees classifiers, (2) PLS-RF [40], which employs partial least-squares-based models, coupled with an RF classifier, (3) MACF [41], which uses a sparse representation technique to recognise a body pose orientation, (4) VGG-16 [42], which uses 16 weighted CNN layers and a CNN with low-resolution images, (5) ResNet-101 [23] based on deep residual nets, (6) the proposed teacher model (proposed T-Model) without handcrafted filters, (7) the proposed teacher model (proposed T-Model), and (8) a combination of the proposed student model with a deep student network and the RF (proposed S-Model). Among the eight comparison methods, methods (4)–(8) are based on a CNN.

Table 1 shows the comparative POE rate of the three conventional approaches and five CNN-based approaches in terms of the average precision (AP), average recall (AR), and average FPR (AFPR).

During all experiments, we confirmed that the CNN-based methods produce better classification performance than the conventional handcrafted and classifier-based methods. Although MoAWG [39] achieved the highest performance among the three conventional approaches, it showed 0.2%, 3.2%, and 0.6% worse values than VGG-16 [42], which are the lowest performances among the five deep-network-based approaches. VGG-16 [42] and ResNet-101 [23] demonstrated a better performance than the conventional approaches [39,40,41], but because they use basic CNN models, we can see that their performance is lower than the proposed method. Among the proposed methods, the proposed T-Model method showed the best performance compared with the other deep-network-based methods in terms of the three measures applied because it uses a teacher network with a teacher RF concurrently.

In the case of the proposed T-Model without handcrafted filters, it showed a lower performance on all three measures as compared with the original T-Model. Based on the results, we can see that a wavelet transform has a good spatial frequency localisation property and can preserve both the spatial and gradient information of an image.

The evaluation results of the proposed S-model show a slightly lower performance of 7.3% and 5.3% in terms of the AP and AR when compared to the T-Model. However, because the performance degradation is small compared with the size reduction ratio of the model, we can infer that the proposed method effectively improves the memory and speed requirements while maintaining the performance. Compared with the other CNN-based methods, the AP and AR are relatively high, and the AFPR is low, which indicates that the proposed method is robust against complex backgrounds or a blurring of the pedestrian outline.

As the second performance evaluation, we presented a confusion matrix to compare the POE classification performance per orientation class for the proposed method based on the level of accuracy. As shown in Figure 3, most of the orientations showed a similar classification performance with the exception of ‘Back’ and ‘Lback’. The main reason for the lower accuracy rate of these two orientations when compared with the other orientations was that the two orientations have a similar appearance, even if the wavelet transform is applied in the previous stage of the CNN. In contrast, the ‘Lfront’ and ‘Rback’ showed the best classification performance owing to the difference in appearance.

### 4.2. Optimal Number of Decision Trees for Student RF

**Figure 4:**
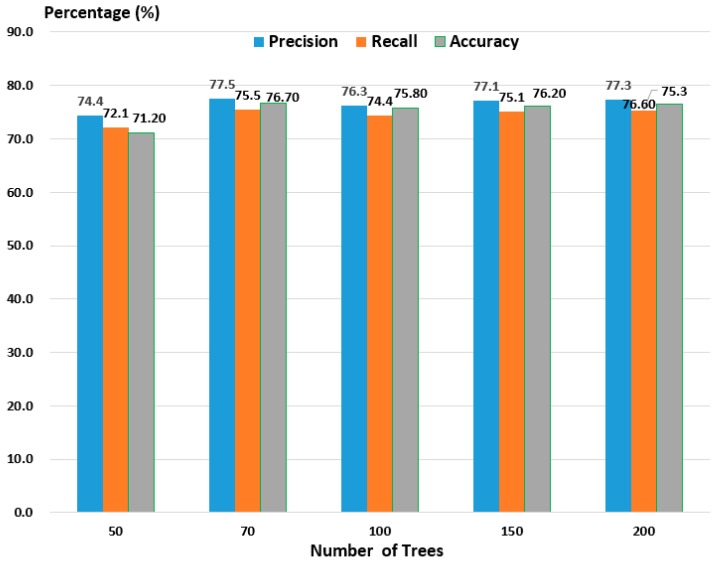
Five possible pairs of experiment results for determining the number of trees for the student RF.

For student RF, the number of decision trees is an important factor in reducing the processing time and the number of parameters for saving memory. To determine the number of optimal trees of the student RF, we compared the precision, recall, and accuracy performance on the TUD dataset while sequentially decreasing the desired number of trees to 200, 150, 100, 70, and 50, as shown in Figure 4.

Figure 4 indicates that, as the number of trees is increased, the precision, recall, and accuracy rates are increased. However, the number of parameters is relatively increased, and the speed and compression rate are decreased. Based on these results, we can see that 70 trees is the optimal number for the RF because it results in a similar or slightly higher performance than the other numbers of trees. Therefore, in this study, we set the number of trees to 70 for better accuracy and a reduced number of parameters.

### 4.3. Evaluation of Model Compression

The goal of the model compression is to generate an optimal student model with a similar performance as the teacher model, but with smaller numbers of parameters and operations. Therefore, we compared the proposed student model with state-of-the-art MobileNet V2 [43] and SqueezeNet V1.1 [44], which are popular model compression methods, in terms of the numbers of parameters and operations using the TUD dataset. The comparison model was fine-tuned using TUD training data based on pre-trained parameters. MobileNet V2 [43] is based on separable depth-wise convolutions applied to reduce the number of parameters and operations. SqueezeNet V1.1 [44] is based on a simple operator called ‘fire module’ to achieve high model capacity and efficiency. In this experiment, four comparison methods were executed using a single Titan-Xp GPU.

As shown in Table 2, the rank of accuracy is *Teacher model* > *Student model* > *MobileNet V2* > *SqueezeNet V1.1*, while the rank on number of operations becomes *SqueezeNet V1.1* > *Student model* > *MobileNet V2* > *Teacher model*. The results show that the POE accuracy of the proposed student method is somewhat lower than that of the deep and wide teacher model, but the numbers of operations and parameters are reduced approximately five-fold required and 19.6-fold. In addition, the proposed student model achieves 17.9% and 25.57% better POE accuracy than MobileNet V2 [43] and SqueezeNet V1.1 [44] respectively, while using a similar number of operations with two compression models. As the comparison results indicate, we can see that the proposed model compression method achieves superior performance in terms of the POE recognition rate and number of operations as compared with two compression methods. However, requiring more parameters than comparative compression models needs to be improved.

### 4.4. Performance Evaluation on KITTI Dataset

To validate whether the algorithm used in the POE can be effectively applied to other datasets, the proposed algorithm was also applied to the KITTI dataset [38], as described in Section 4, and the results were compared. For the training of the KITTI dataset, we used 5415 images including pedestrians, and a validation set consisting of 2065 images was applied for testing. A performance evaluation was conducted based on difficulties of the dataset that have been divided into three levels according to their sizes, occlusions, and truncation levels, namely, *easy*, *moderate*, and *hard*. We used the average orientation similarity (AOS) for the KTTI dataset to measure the difference in continuous orientation.

For the performance evaluation, we compared the accuracy against the following state-of-the-art methods: (1) DPM-VOC+VP [45], which extends the deformable part of the model scheme to handle different viewpoints, (2) Mono3D [46], which detects 3D objects from a single monocular image using a CNN, (3) SubCNN [47] based subcategory-aware convolutional neural networks, (4) FRCNN [48] using a viewpoint inference on top of a highly optimised CNN-based detection framework, and (5) the proposed student model.

Table 3 summarises the experimental results based on the evaluated KITTI POE classification on five CNN-based methods. In the experiment using the KITTI dataset, the two comparison methods, DPM-VOC+VP [45] and FRCNN [48], demonstrated a lower AOS rate than the other three methods when the input image is small owing to a blurring of the outer edges of the pedestrian through the frequent convolutions and pooling operations of the deep CNN. However, the SubCNN [47] method achieves a better AOS performance than other methods in terms of the three levels because it uses an image pyramid to detect small-sized pedestrians. Although SubCNN [47] showed a relatively good AOS rate for the KITTI dataset, it has a disadvantage in that the network structure is deeper and wider than that of the proposed algorithm, and it requires an additional network for region proposal and object detection. However, the proposed method improves the AOS rate by applying a teacher–student structure and the two compressed classifiers (student network and student RF) complement the disadvantages of the other, thus demonstrating a good performance for the *hard*, *easy*, and *moderate* data of the KITTI dataset with various changes to the background.

**Figure 5:**
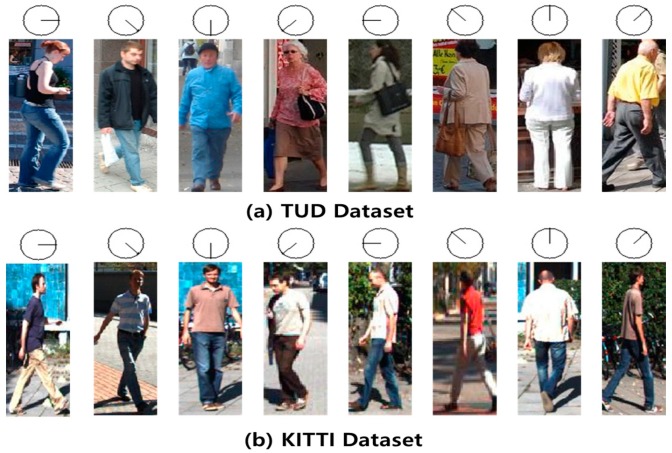
Sample orientation classification results for TUD and KITTI datasets using the proposed method: (**a**) estimation of pedestrian’s body orientation using TUD dataset, (**b**) results of pose orientation estimation (POE) using KITTI.

Figure 5 shows the POE results of the TUD (Figure 5a) and KITTI (Figure 5b) datasets using the proposed student model. As shown in Figure 5a,b, the proposed approach predicts the pedestrian’s orientation correctly when the pedestrian’s body is distorted or partly occluded by other pedestrians, and even if the image is blurry.

## 5. Conclusions

In this study, we proposed a new POE method for application in a real driving situation using the proposed framework, which consists of a teacher model and a student model. The teacher model generates the probability values for each class by combining the output of the teacher network and teacher RF and trains the student model by inputting these soft target values. By combining two different classification models, we not only can reduce the model size but also construct a student network capable of mimicking the POE capability of the teacher model.

Unlike conventional CNN-based POE approaches, this study chooses a new soft output by combining the softened output of the teacher network and the teacher RF and constructs a shallower student model with an equal performance based on the teacher model.

The experimental results using two benchmark datasets showed that our algorithm improves the POE performance compared to other state-of-the-art methods based on conventional classifiers as well as a CNN. Moreover, it was proved that the proposed student model requires a small amount of memory and fewer operations as compared to deeper and shorter CNN-based networks and teacher models. Therefore, we confirmed that the proposed POE method is applicable not only to the embedded systems of intelligent vehicles but also for use in various other fields, such as surveillance and robot vision.

As a future study, we plan to improve our algorithm to reduce false predictions when a pedestrian is occluded by other objects or when the shape of the pedestrian is severely deformed. In particular, because the prediction speed is also a significant factor for real-time applications, our research will focus on developing a faster student framework while maintaining a higher accuracy than the current framework. In addition, we will improve the teacher–student model that can recognize pedestrian POE robustly to various environment changes (e.g., day and night, illumination change) with a single POE recognition algorithm by developing an efficient image-to-image image translation method based on generative adversarial networks [49] as the pre-processing.

## Figures and Tables

**Table 1:** Performance comparison results on eight methods (We referred to the results of the first three methods from the experimental evaluations in [19]).

Methods	AP (%)	AR (%)	AFPR (%)
MoAWG [39]	67.4	65.4	4.9
PLS-RF [40]	66.3	62.3	5.0
MACF [41]	41.1	40.0	8.5
VGG-16 [42]	67.6	68.6	4.3
ResNet101 [23]	73.5	74.6	3.9
Proposed T-Model without handcraft filters	75.4	76.8	3.1
Proposed T-Model	85.6	84.6	2.0
Proposed S-Model	**78.3**	**79.3**	**2.9**

**Table 2:** Comparison between the numbers of parameters and operations for the proposed method and two state-of-the-art compression models using the TUD dataset.

Methods	Accuracy (%)	No. of Parameters (M)	No. of Operations (M)
MobileNet V2 [43]	60.73	2.2	430
SqueezeNet V1.1 [44]	53.06	0.72	283
Teacher model	85.08	47.2	7564
Proposed student model	**78.63**	**9.5**	**385**

**Table 3:** Comparison of the average orientation similarity (AOS) for the proposed method and a deep neural network-based approach using the KITTI dataset. (We referred to the results of four comparison methods from the experimental evaluations in [44]).

Methods	Average Orientation Similarity (AOS, %)		
	*Easy*	*Moderate*	*Hard*
DPM-VOC+VP [45]	53.66	39.83	35.73
Mono3D [46]	68.58	58.12	54.94
SubCNN [47]	78.33	66.28	61.37
FRCNN [48]	66.84	52.62	48.72
Proposed student model	**81.1**	**68.61**	**63.54**
